# High-throughput physical map anchoring via BAC-pool sequencing

**DOI:** 10.1186/s12870-015-0429-1

**Published:** 2015-04-11

**Authors:** Kateřina Cviková, Federica Cattonaro, Michael Alaux, Nils Stein, Klaus FX Mayer, Jaroslav Doležel, Jan Bartoš

**Affiliations:** Institute of Experimental Botany, Centre of Region Haná for Biotechnological and Agricultural Research, Šlechtitelů 31, 78371 Olomouc-Holice, Czech Republic; Istituto di Genomica Applicata, Via J. Linussio 51, 33100 Udine, Italy; INRA, UR1164 URGI - Research Unit in Genomics-Info, INRA de Versailles, Route de Saint-Cyr, 78026 Versailles, France; Leibniz Institute of Plant Genetics and Crop Plant Research, Corrensstraße 3, 06466 Stadt Seeland, OT Gatersleben Germany; Plant Genome and Systems Biology, Helmholtz Zentrum München, 85764 Neuherberg, Germany

**Keywords:** Physical map, Contig anchoring, Next generation sequencing

## Abstract

**Background:**

Physical maps created from large insert DNA libraries, typically cloned in BAC vector, are valuable resources for map-based cloning and *de novo* genome sequencing. The maps are most useful if contigs of overlapping DNA clones are anchored to chromosome(s), and ordered along them using molecular markers. Here we present a novel approach for anchoring physical maps, based on sequencing three-dimensional pools of BAC clones from minimum tilling path.

**Results:**

We used physical map of wheat chromosome arm 3DS to validate the method with two different DNA sequence datasets. The first comprised 567 genes ordered along the chromosome arm based on syntenic relationship of wheat with the sequenced genomes of *Brachypodium*, rice and sorghum. The second dataset consisted of 7,136 SNP-containing sequences, which were mapped genetically in *Aegilops tauschii,* the donor of the wheat D genome. Mapping of sequence reads from individual BAC pools to the first and the second datasets enabled unambiguous anchoring 447 and 311 3DS-specific sequences, respectively, or 758 in total.

**Conclusions:**

We demonstrate the utility of the novel approach for BAC contig anchoring based on mass parallel sequencing of three-dimensional pools prepared from minimum tilling path of physical map. The existing genetic markers as well as any other DNA sequence could be mapped to BAC clones in a single *in silico* experiment. The approach reduces significantly the cost and time needed for anchoring and is applicable to any genomic project involving the construction of anchored physical map.

**Electronic supplementary material:**

The online version of this article (doi:10.1186/s12870-015-0429-1) contains supplementary material, which is available to authorized users.

## Background

Physical maps are important tools for genomic studies both in animal and plant species. Among other, they facilitate positional gene cloning in crop plant species [1,2]. Thus, cloning of at least thirteen genes is currently underway in hexaploid wheat (*Triticum aestivum*), making use of physical contig map [3]. Apart from positional gene cloning, physical maps have been used in genome sequencing projects [4]. Although a majority of higher plant genomes have been sequenced by whole genome shotgun strategy [4], hierarchical approach of genome sequencing with the intermediate in form of physical contig map anchored to individual chromosomes is a prerequisite to obtain high-quality reference sequences [5,6]. Physical contig maps are typically constructed from bacterial artificial chromosome (BAC) libraries that are created from genomic DNA digested by restriction enzymes and cloned in a BAC vector [7]. After BAC library construction, High-Information Content Fingerprinting (HICF) technology [8] detects clone overlaps on the basis of sharing restriction spectrum [9] and fingerprinted data are statistically elaborated by Finger Printed Contigs (FPC) [10,11] or Linear Topological Contig (LTC) [12] software. A physical map consists from BAC clones organized into contigs (sets of overlapping clones) whose number depends on genome coverage and insert size of BAC clones.

The utility of physical maps for positional gene cloning and genomic studies is limited until the contigs of BAC clones are ordered along chromosomes, usually with the help of high density genetic maps. An indispensable step in integrating physical and genetic maps is the screening of BAC library, which assigns molecular markers to individual BAC clones. The screening has been done either by PCR with marker-specific primers [13], or by hybridization of markers to BAC DNA spotted on a filter [14]. Historically, BAC library screening was the most laborious and expensive step in constructing anchored physical map. The procedure has been gradually improved to achieve more effective anchoring of a majority of contigs. A BAC library could be screened using multidimensional pooling strategy [15,16]. A BAC pool is prepared by combining predefined set of BAC clones within or between 384-well plates in which the clones are stored [16]. Three-dimensional pooling strategy (plate, row, and column pools) [15,16] has been the most popular approach, which could be further improved by adding superpools to minimize the number of PCR reactions needed to link a marker with a BAC clone [17]. To simplify and speed up the anchoring process, it is also possible to use methods like multiplex tandem PCR with high resolution melt analysis [13], microarray platforms [18], or Illumina GoldenGate assay [19]. All these techniques avoid gel electrophoresis, which is most laborious part of PCR screening and cannot be done in a high-throughput manner.

Another critical aspect in contig anchoring is the availability of a high-density genetic map with a sufficient number of molecular markers covering evenly the whole genome. Such maps are becoming available for a growing number of species [20-22] thanks to progress in methodology and instrumentation of molecular biology and genomics, which resulted in negligible cost per data point. However, general problem of genetic maps is a poor resolution in centromeric and pericentromeric regions due to the lack of recombination. In fact, poorly recombining regions may represent 40% of chromosome as show in barley [23]. Alternative approaches have been developed to organize molecular markers along the chromosome independently on meiotic recombination, and included deletion bin mapping [24], HAPPY mapping [25] and radiation-hybrid mapping [26]. A recent addition is the approach called “GenomeZipper” [27]. GenomeZipper is a bioinformatic pipeline constructing a virtual gene order in a particular genome through comparative analysis using synteny conservation with species already sequenced. For crops belonging to tribe *Triticeae*, *Brachypodium* [28], rice [29] and sorghum [30] are typically used to order genes along chromosomes [31-33].

The crops in tribe *Triticeae* are characterized by large and complex genomes. Bread wheat (*T. aestivum*), one of the three major crops worldwide has hexaploid (2n = 6x = 42) genome of 17 Gbp, which comprises three closely related sub-genomes and contains more than 90% repetitive DNA. Special approach called “chromosome based genomics” has been developed to handle physical mapping and sequencing of the wheat genome [34]. BAC libraries have been constructed from DNA of flow sorted chromosomes and chromosome arms [35,36]. The availability of BAC libraries from individual chromosomes greatly simplifies the construction of ready to sequence physical maps and the analysis of the complex allohexaploid wheat genome.

Here we present novel approach for BAC library screening and contig anchoring based on Illumina sequencing of three-dimensional BAC pools prepared from minimum tilling path (MTP). Genetic markers as well as any other sequences can be mapped easily to BAC clones in a single *in silico* experiment. We used wheat chromosome arm 3DS to demonstrate the utility of our novel approach by anchoring about 750 sequences of intra- and inter-specific origin to the physical contig map.

## Results and discussion

**Figure 1:**
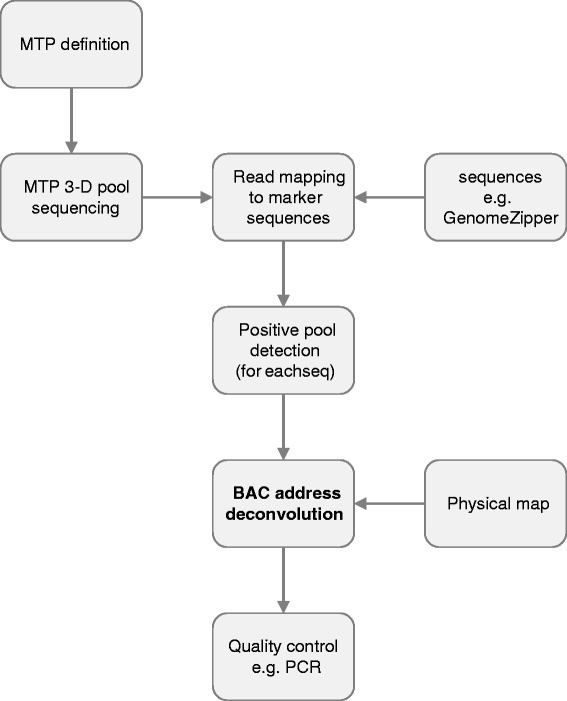
Graphical overview of the procedure for *in silico*physical map anchoring.

Ordered physical contig maps are valuable resources for genome analysis, production of reference sequences of complex genomes, and positional gene cloning. However, efficient use of physical maps requires that clone contigs are anchored to chromosomes and ordered along them using molecular markers. The aim of the present work was to develop *in silico* procedure for BAC contig anchoring. The approach we have validated makes screening of BAC library cost effective and more flexible. The procedure includes mas parallel sequencing three dimensional BAC pools, mapping sequence reads to marker sequences, positive pool identification and BAC address deconvolution (see Figure 1).

### BAC-pool sequencing

**Figure 2:**
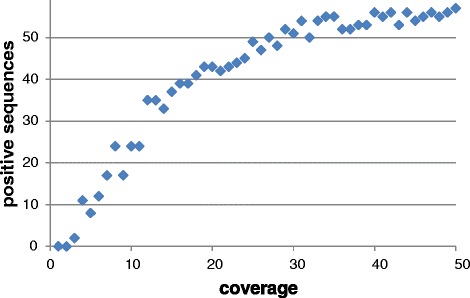
Coverage test for read alignment. Reads representing different sequencing depth of BAC pool rP from 1x to 50x were randomly selected from sequencing data and aligned to 567 sequences represented in GenomeZipper dataset. Sequences covered with aligned reads at least from 80% of the length were considered positive (i.e. represented in the pool). The curve reached plateau at about 30x coverage. The noise of the growth curve is due to random selection of reads for alignment.

The original target for the sequencing was at least 10x coverage for each of BAC pools. Finally, about 180 million reads were generated in three Illumina HiSeq2000 lines. Mean coverage of BAC pools reached nearly 35x. However, the coverage ranged from 5.9 for pool p09 to 166.5 for pool rP (for complete statistics see Additional file 1). So large differences in coverage of BAC pools were not expected. To investigate the effect of sequencing depth, we selected and mapped reads representing different depth of pool rP from 1x to 50x to GenomeZipper sequence dataset and counted the sequences identified in the pool. This parameter reached a plateau at 30x coverage (Figure 2).

### Read alignment optimization

**Figure 3:**
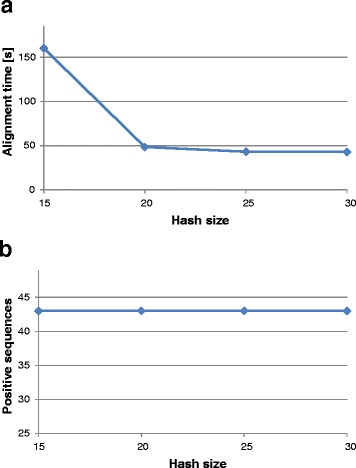
Hash size test for read alignment. Hash size was tested with respect to alignment sensitivity and time needed for analysis. **a**) Time needed to align 30x coverage of pool rP to 567 sequences in GenomeZipper dataset. **b**) The number of sequences positive for pool rP. Sequences covered with aligned reads at least from 80% of the length were considered as positive (i.e. represented in the pool). While hash size has no effect on alignment sensitivity, increasing hash size reduced time needed to align reads.

Reads of BAC pool rP were aligned using Mosaik to GenomeZipper sequence dataset with the aim to optimize alignment parameters. The BAC pool was considered positive for a particular sequence if “covered region” (length of the sequence region covered by reads from the pool) was at least 80% of the sequence length. Hash size (k-mer length for alignment) was optimized first without any limitation of alignment candidate threshold. Time needed for read mapping, decreased dramatically for hash size between ten and fifteen (from ~ 10 hours to 160 seconds). Further decrease of computation time is shown in Figure 3a. The sensitivity of alignment was not influenced by hash size, and the number of sequences found in the pool rP remained 43 for any hash size used (Figure 3b). Considering these results, hash size 20 was selected for further analysis.

**Figure 4:**
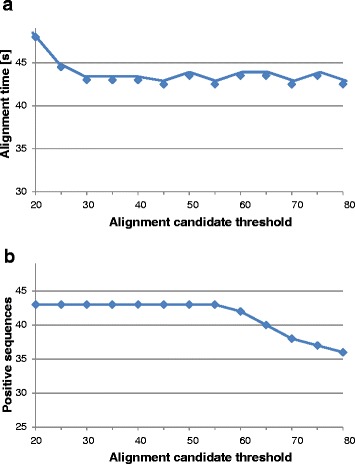
Alignment candidate threshold test for read alignment. Alignment candidate threshold was tested with respect to alignment sensitivity and analysis time. **a**) Time needed to align 30x coverage of pool rP to 567 sequences represented in GenomeZipper dataset. **b**) The number of sequences positive for pool rP. Sequences covered with aligned reads at least from 80% of the length were considered as positive (i.e. represented in the pool). While alignment candidate threshold has negligible effect on alignment sensitivity, increasing hash size reduced number of positive sequences as it is getting closer to read length (100 bp).

Alignment candidate threshold (minimal alignment length to map the read) was optimized with hash size 20. When the alignment candidate threshold is set, only alignments reaching at least the set length are considered. We tested this parameter for values between 20 and 80. In parallel to hash size, time needed for the analysis decreased with the increased alignment candidate threshold. However, the effect was negligible when compared to that for hash size (Figure 4a). The number of positive sequences found in the pool ranged from 43 to 36 (Figure 4b) and decreased with alignment candidate threshold getting close to 100 bp (read length). However, the sensitivity of sequence alignment remained stable for alignment candidate thresholds up to half of the read length in our case (50 bp). Alignment candidate threshold 40 was selected for further analysis as there was no reduction in the analysis time if it was further increased, and this value was low enough to keep the expected alignment sensitivity.

The pool rP should be positive for 6.3% of sequences (i.e. 35 sequences) as it was one of sixteen row pools. Thanks to overlaps of BAC clones in the MTP, each row pool represents more than 6.3% of entire physical map. On the other hand, one cannot expect that physical map represents the complete chromosome arm as it may carry regions lacking restriction site for the enzyme used to construct the BAC library. Overall, the 43 positive sequences out of 567 for pool rP agreed well with our expectation.

### Read alignment

The criteria optimal for row pool rP (see above) were used for all other pools: hash size 20; alignment candidate threshold 40. The number of positive sequences per BAC pool (with covered region ≥ 80%) was counted after the final read alignment. Alignment of reads of individual BAC pools to 567 GenomeZipper sequences resulted in 10 to 62 positive sequences per pool. On average, 43.4, 38.4 and 27.0 sequences were aligned to plate, row and column pools, respectively (for details see Additional file 1). The smallest number of positive sequences was found in plate pool p09, which was significantly under-sequenced (5.9x only). A distribution of sequences in pooling dimension (plates, rows and columns) showed reduced detection of sequence markers in plate pools. On average, only 11.3 sequences per 100 BAC clones were detected in plate pools, 16.0 sequences per 100 clones in row pools and 16.9 sequences per 100 clones in column pools. This phenomenon could reflect larger growth differences among 384 clones in plate pools as compared to 240 and 160 clones in row and column pools, respectively. Under-representation of a particular clone (containing the target sequence) in the pool could then lead to false negative result for the pool after read alignment.

Twenty nine to ninety five positive sequences per BAC pool (out of 7,136) were identified in *Ae. tauschii* sequence dataset (for details see Additional file 1). Mean values were 75.1, 65.7 and 49.0 positive sequences per plate, row and column pool, respectively. As for the GenomeZipper dataset, plate pool p09 had the smallest number of positive sequences, and plate pools showed the lowest number of detected sequences. On average, 19.6, 27.4 and 30.6 sequences were detected in 100 BAC clones in plate, row and column pools, respectively. Pools with sequencing depth lower than twenty were more likely to have lower number of positive sequences (see Additional file 1). These observations suggest that minimal coverage for each pool should be 20. Otherwise, increased frequency of false negative results for under-sequenced pools (sequence is not scored in the pool if it is physically present) can lead to reduced number of anchored sequences.

### Positive pool detection

**Figure 5:**
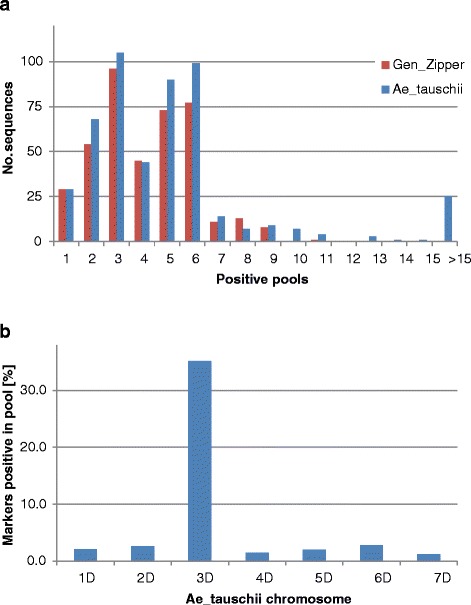
Positive pool detection. Each individual pool was considered positive, if its reads covered at least 80% of particular sequence. **a**) Distribution of the number of sequences positive for given number of pools for GenomeZipper and *Ae. tauschii* sequence dataset. Only sequences with at least one positive pool were considered. **b**) Distribution of sequences with at least one positive pool among seven *Ae. tauschii* chromosomes. Note that a majority of sequences originate from chromosome 3D.

Alignment of reads from individual BAC pools to GenomeZipper sequence dataset resulted in a variable number of positive pools per individual sequence (Figure 5a). 407 (71.8%) GenomeZipper sequences were found in at least one pool and the remaining 160 sequences were not scored in any of the fifty pools. To explain this, we screened the pools with primers specific for ten of the sequences using PCR. Out of ten markers, eight identified at least one positive pool after PCR screening the pools (data not shown), which were prepared in the same way as for sequencing. This indicates high level of false negative results. As mentioned above, sequencing depth could influence the identification of pools containing target sequences. Thus, the pools with lower sequence depth could be more frequently false scored as negative. Further, individual clones in pools could be under-represented in the sequence reads, and hence not covering particular sequence by reads enough to reach the threshold. Finally, duplicated regions among sequences with 100% identity could not be covered by any read as only reads mapping to unique positions were used for the analysis.

Similarly to GenomeZipper dataset, alignment of reads from BAC pools to *Ae. tauschii* sequence dataset resulted in a variable number of positive pools per sequence (Figure 5a). Excessive number of pools was positive for several sequences, and for three sequences even all fifty pools were positive (all three represent transposable elements). This fact led to the modification of BAC address deconvolution script and all markers with more than five positive pools in any of the dimensions (plate, row, column) were considered repetitive and were not assigned to any of the BAC clones detected by the script. Out of the 7,136 *Ae. tauschii* sequences, 506 (7.1%) were detected in at least one BAC pool. While GenomeZipper was constructed specifically for 3DS chromosome arm, *Ae. tauschii* sequences originate from all seven chromosomes. This led to lower fraction of sequences detected in pools as compared to the GenomeZipper dataset. On the other hand, it allowed us to screen pools for sequences originating from other genome regions and thus estimate the rate of false positive results. For non-target chromosomes (i.e. 1D, 2D, 4D, 5D, 6D and 7D) the frequency of sequences scored in at least one pool ranged from 1.2% for chromosome 7D to 2.8% for chromosome 6D (Figure 5b). These results indicate low level of false positive results, which we estimated below 3%. 35.2% sequences genetically mapped to *Ae. tauschii* chromosome 3D were detected in pools. The ratio of molecular sizes of the short and long arm of 3D chromosome can be used to estimate the number of markers on the short and long arm. Using the ratio 321:449 Mbp [37], 455 *Ae. tauschii* sequences with up most positions on genetic map should correspond to 3DS chromosome arm. Among them, 370 (81.3%) were indeed found in at least one pool. Out of the markers mapped to the long arm of *Ae. tauschii* chromosome 3D, only 14 (1.9%) were positive in BAC pools from wheat 3DS.

### BAC address deconvolution

**Figure 6:**
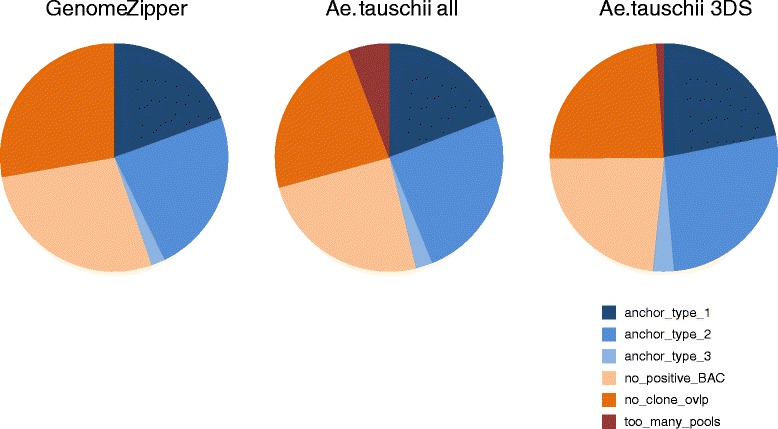
Anchoring results for sequences with at least one positive pool. For both sequence datasets (GenomeZipper, *Ae. tauschii*), about half of sequences were anchored to BAC clones/physical map contigs. A particular BAC pool was considered positive if its reads covered at least 80% of particular sequence after read alignment to complete sequence set. A majority of the remaining sequences were not anchored either due to missing positive pool in one of the dimensions (no_positive_BAC) or no candidate clone overlap during the deconvolution (if more than one clone is positive; no_clone_ovlp).

**Figure 7:**
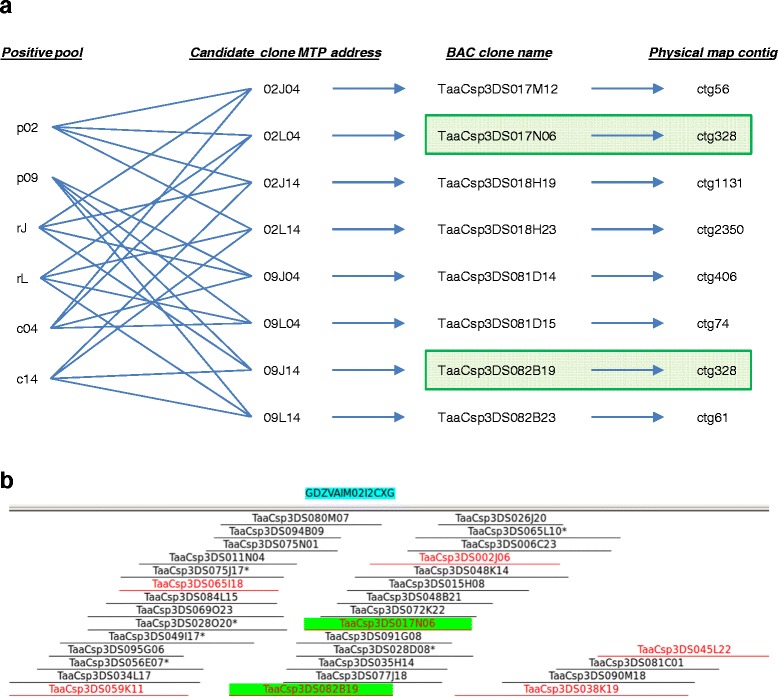
Examples of deconvolution for multiple positive pools in each pooling dimension. **a**) Eight candidate clones exist for six positive BAC pools (2 plates, 2 rows and 2 columns). MTP address of candidate clones is first translated to BAC clone name and contig name for each clone is then retrieved from the physical map. If two clones among the candidates share the same contig (TaaCsp3DS017N06 and TaaCsp3DS082B19 in this example), they are considered positive. **b**) The image of contig ctg328. Note overlap of clones TaaCsp3DS017N06 and TaaCsp3DS082B19 (labelled in green) detected as positive ones in part a).

Each BAC clone is present in a single plate, row and column pool (e.g. BAC clone at position F17 in MTP plate no. 3 is present in pools p03, rF and c17; see Methods for description of BAC pool preparation). Thus, it should be possible to deconvolute positive pools to individual clones using the information about the presence of individual clones in pooled samples. In other words, each combination of single plate, row and column pools could be unambiguously deconvoluted to a single BAC clone. Thus, it was possible to link sequences with one positive pool in each dimension to a particular BAC clone (anchor_type_1). This type of sequences represented 19.4% and 19.2% of all sequences scored in at least one pool using GenomeZipper and *Ae. tauschii* datasets, respectively (Figure 6). For sequences with more than one positive pool in any of the dimensions, positive clones were selected from all possible candidates. For example, in case of two positive plate pools, two positive row pools and two positive column pools, there are eight (2x2x2) candidate clones. As all positive clones contain the same unique sequence, they should overlap, at least partially. We used the information from the physical map to verify this. First, if two or more clones among the candidate clones were in the same contig in the physical map, they were selected as positive clones (anchor_type_2, Figure 7). Second, information about putative clone overlap at relaxed cutoff (compared to contig building) was utilized. Two or more clones were selected as positive out of the candidates if they were placed at the ends of BAC contigs (end clones) and they matched each other at cutoff 1e-25 (anchor_type_3). These two approaches resulted in anchoring 95 (23.3%) and 8 (2%) sequences from GenomeZipper dataset. Using the same strategy for *Ae. tauschii* dataset, 125 (24.7%) and 12 (2.4%) sequences were anchored to physical map contigs. Altogether, 182 (44.7%) sequences from GenomeZipper and 234 *Ae. tauschii* datasets were linked with physical map contigs. Within the *Ae. tauschii* dataset, 191 sequences were assumed to originate from chromosome arm 3DS representing 42.0% of sequences mapped genetically to chromosome arm 3DS. After the deconvolution, some sequences remained not anchored as no positive BAC clone was identified for them even if they were positive for at least one pool. Majority of them were represented by sequences with a missing positive pool in at least one of the dimensions (plate, row or column; unequivocal deconvolution is not possible in such case) and sequences for which no overlap/match of any two candidate clones was identified in physical map (for details see Figure 6). For those sequences, false negative results for some pools prevented identification of positive BAC clones.

### Analysis with decreasing stringency of positive pool detection

Based on the results of the initial analysis (lower number of anchored sequences, tendency to false negative results), we performed the analysis with successively lower value for covered region (region covered by reads from particular pool). We decreased the covered region from 80% to 30% of sequence length in steps of 10% (see Additional file 2 for complete datasheet). With the GenomeZipper dataset, the threshold 30% of covered region resulted in at least one positive pool for 541 (95.4%) sequences. The increased number of sequences found in pools confirmed false negative results obtained after the initial stringent analysis, which was indicated by PCR screening (see above). 441 sequences from GenomeZipper dataset could be anchored to BAC clones based on less stringent criteria (30% of length of particular sequence covered by pool reads). This represented more than twofold increase in the number of anchored sequences as compared to more stringent conditions (covered region 80%). Similarly, 60% increase of anchored sequences (from 191 to 305) was achieved when the same conditions of anchoring were applied to *Ae. tauschii* sequences mapped to chromosome arm 3DS (see Additional file 3 for complete statistic at successive lower stringencies). After the analysis at several successive lower covered regions, we compared anchoring results between the most and least stringent analysis. Among 182 GenomeZipper sequences anchored at covered region 80%, 165 were anchored to the same contig with covered region 30%. This indicates the robustness of the pipeline. In case of six sequences, identification of additional positive pools under less stringent conditions resulted in identification of different clones/contigs after BAC address deconvolution. For the remaining eleven markers, additional positive pools prevented unambiguous identification of positive BAC clones. We used PCR to confirm the presence of particular sequence in clones detected using either high or low stringency conditions. In four cases (sequences GDZVAIM01DAD8G, GDZVAIM01EPQJO, GDZVAIM02GXHAN, GDZVAIM02H0YGY), BAC clones identified under low stringency conditions (detecting more positive pools/clones) were positive after PCR with specific primers. In one case (GDZVAIM01EDIJF), clones under both high and low stringency conditions of analysis were found positive after PCR screening. For the last sequence (GDZVAIM02I33VW) no clone identified after BAC address deconvolution in *in silico* analysis was positive (for details see Additional file 4). All these results indicate a tendency for false negative pool detection rather than for false positive pool detection.

### Anchoring quality control

After the analysis with a decreasing stringency, we used PCR to verify the results of *in silico* analysis. We randomly selected fifteen representatives of anchored sequences (five anchor_type_1, five anchor_type_2 and five anchor type_3) and used specific primers to confirm the presence of sequences in particular clone(s). In all fifteen cases, PCR confirmed the presence of particular sequences in clones detected *in silico* (for details see Additional file 4). To further confirm the anchoring results, we investigated all physical map contigs with two and more sequences anchored and assessed the distance of anchored sequences based on their order in the virtual map (GenomeZipper). Out of the 100 BAC contigs with multiple GenomeZipper sequences, a majority (92%) contained neighbouring genes in GenomeZipper. In eight contigs, genes more distant in GenomeZipper were found (separated by at least five additional genes in GenomeZipper). Some of these genes could be anchored to incorrect contigs. However, there are at least four explanations: 1) chimeric BAC clones; 2) missassembly of physical map contigs; 3) incorrect order of genes in GenomeZipper; and 4) false anchoring of sequences to particular contigs. Similarly, we checked the genetic distance of *Ae. tauschii* sequences anchored to one physical map contig. We found 59 contigs with more than one sequence genetically mapped to chromosome 3D of *Ae. tauschii*. In 54 cases (92%), genetic distance of particular SNPs was lower than 1 cM.

### Utility of BAC pools sequencing for physical map anchoring

We were able to anchor 311 sequences from *Ae. tauschii* dataset genetically mapped to chromosome arm 3DS using combination of high and low stringency anchoring conditions (see Additional file 5 for complete sequence list and their BAC/contig addresses). Under the same conditions, 447 sequences represented in 3DS GenomeZipper dataset were assigned to physical map contigs. This represent 78.8% of 567 non-redundant gene fragments (454 reads) organized in wheat 3DS GenomeZipper (see Additional file 5 for complete sequence list and their BAC/contig addresses). Recently, Poursarebani et al. [38] used similar approach for physical map anchoring. Authors used short sequence tags produced from whole genome profiling (WGP) [39] to anchor sequences to physical map of wheat chromosome 6A. They were able to anchor 67% genes used for anchoring. In comparison, we successfully anchored 79% gene fragments. While reads produced after BAC pools sequencing are distributed evenly along the whole BAC insert, sequence tags are produced from specific sites surrounding recognition pattern of restriction enzyme used for WGP. Consequently, missing restriction site in the region of a gene could make anchoring of that particular gene impossible. Poursarebani et al. [38] used for physical map building BAC clones with 6–68 sequence tags. It is likely, that clones with low number of sequence tags could more frequently miss a tag in the regions of genes they contain. Clear advantage of the method used by Poursarebani et al. [38] over our approach is the absence of additional cost after the physical map building. However, BAC clone fingerprinting using whole genome profiling is not the only possibility for physical map construction and may not be always the best possibility. As an alternative, HIFC technique [8] for BAC fingerprinting could be used and was used many times. No sequence tags are produced by HIFC technique and physical map cannot be anchored by approach used by Poursarebani et al. [38].

Hybridization of pooled BAC-DNA with wheat 40 k unigene array [40] was recently used to anchor genes to several wheat physical maps of wheat chromosome arms [41-43]. For the wheat chromosome 1A, 755 and 1,231 genes were placed on the physical map of short and long arm, respectively [41,42]. Genes syntenic with *Brachypodium distachyon* represent about one third of those genes (254 and 381). Here we anchored a comparable number of syntenic genes with *B. distachyon* (non-collinear genes were not used through whole analysis). Compared to physical map of chromosome arm 1AL, we reached higher efficiency of gene positioning. While Lucas at al. [42] were able to anchor 381 (28%) out of 1,352 syntenic genes or gene fragments, we were able to found BAC address for 79% of syntenic genes. The advantage of our approach is a possibility to change stringency of anchoring conditions. In case of array hybridization, this means re-hybridization of BAC-DNA with the array under changed conditions, which increases the cost of experiment. Once BAC pool sequencing is done, there are no additional expenses for *in silico* anchoring using different parameters. Philippe et al. [43] used array hybridization to anchor ISBP (insertion site based polymorphism) markers. They succeed in anchoring of 3,912 ISBP markers to physical map of wheat chromosome arm 1BL. However, the experiment again required hybridization of all pooled BAC-DNA samples with an array. As the ISBP markers are a popular type of marker, we tested the utility of *in silico* anchoring also for this type of markers and found that many repetitive sequences, which are not present in high copy number in wheat genome could be assigned to individual BAC clones (data not shown). In the present study we tested *in silico* anchoring with a relatively small number of sequences. Nevertheless, 390 contigs were anchored to 3DS chromosome arm by at least one marker, and these contigs represent 36.1% physical map length (110 Mbp). We expect that a majority of contigs could be anchored in a single experiment as additional sequence resources have recently become available for hexaploid wheat [44,45].

## Conclusion

The first step after BAC contig building is anchoring the contigs to genetic map as it increases the utility for physical map based cloning and genomic studies. Here we present novel approach for contig anchoring based on mass parallel sequencing 3-dimmensional BAC pools prepared from MTP of physical map. We demonstrate that genetic markers as well as other sequences can be easily mapped to BAC clones in a single *in silico* experiment. We used physical contig map of wheat chromosome arm 3DS in a pilot experiment to validate the utility of this approach by anchoring 758 sequences of intra- and inter-specific origin. The approach described in present study could significantly reduce anchoring costs and time needed and is applicable to any genomic project aiming at constructing anchored physical map. The only prerequisite is the availability of sequenced markers ordered along the chromosome. However, such markers can be easily obtained by mass parallel sequencing and linearly ordered through the comparative analysis and synteny conservation with the sequenced model species [27].

## Methods

### Preparation of DNA pools

BAC library specific for wheat chromosome arm 3DS (TaaCsp3DShA; [37]) was used to validate the BAC pool sequencing strategy for physical map anchoring. The library comprises 36,864 clones with mean insert size 110 kbp. A physical map was build using HIFC technique [8] in Fingerprinted Contigs software (FPC V9.3; [11]) following “physical map assembly guideline” established by TriticeaeGenome project and accepted by the International Wheat Genome Sequencing Consortium (IWGSC). Briefly, each clone was fragmented using a cocktail of five restriction enzymes. Fragment ends produced by four of them were labelled by one of four different fluorescent dyes. DNA fragments were subsequently analysed using capillary electrophoresis, and physical map was built based on the number of shared fragments (with the same size and label) among clones at stringency cutoff 1e-75 to produce robust contigs. The cutoff represents significance threshold for clone overlap and it is based on a probability that two clones share particular number of fragments by chance. If two clones share enough fragments, the probability of random similarity is below the given threshold and the clones match each other and overlap within one contig. Singletons were then added to existing contigs and contigs were merged at successively increasing cutoff up to 1e-45. The complete guideline is available at http://www.wheatgenome.org/News-and-Reports/Meetings-and-Workshops/Physical-mapping-standard-protocol-workshop. Finally, the physical map consisted of 1,360 contigs built using a cutoff of 1e-45 (Cviková et al., unpublished, https://urgi.versailles.inra.fr/gb2/gbrowse/wheat_phys_3DS_v1/). At this stage, MTP was selected corresponding to 3,823 BAC clones re-ordered into ten 384-well plates. Three-dimensional pooling strategy was selected for DNA preparation in an effort to reduce sequencing costs [15]. In total, fifty BAC pools were constructed (10 plate pools, 16 row pool, and 24 column pools) as follows. Each of ten plate pools (labelled p01 – p10) was prepared by pooling all 384 BAC clones deposited in one of the ten MTP plates. Each of sixteen row pools (labelled rA – rP) was prepared by pooling 240 BAC clones deposited in a particular row (e.g. row A) in all ten plates. Similarly, each of 24 column pools (labelled c01 – c24) was prepared by pooling 160 BAC clones deposited in a particular column (e.g. column 1) in all ten plates. BAC clones of each pool were transferred using GeneTAC G^3^robot (Genomic Solutions, Huntingdon, UK) onto solid 2YT medium and incubated for 16 hours at 37°C. Bacterial colonies were then washed to liquid 2YT medium and incubated for 8 hours at 250 rpm and 37°C. DNA was isolated by standard alkaline lysis with minor modifications.

### BAC-pool sequencing

2.5 μg DNA of each of 50 BAC pools was randomly fragmented in 5 μl Fragmentase (New England Biolabs, Hitchin, UK) at 37°C for 5.5 hours. Paired-end libraries were prepared for each BAC pool as recommended by Illumina for multiplexing using the NEB-Next modules from New England Biolabs and oligonucleotide primers for enrichment-PCR with six base indexes. Equimolar amounts of each library were pooled, gel-recovered in the fraction 400-600 bp and sequenced on Illumina HiSeq2000 with 100 cycles per read. To test the read mapping performance, reads representing desired coverage of pool rP were randomly selected. Data for individual BAC pools were reduced to the maximum of 30x coverage if needed prior final read mapping experiment.

### Sequences used for the mapping

We mapped two different sequence datasets to BAC clones from MTP of 3DS arm. The first was produced using GenomeZipper pipeline [27]. Briefly, the chromosome arm was flow-sorted, its DNA amplified according to Šimková et al. [46] and sequenced using Genome Sequencer FLX. One full run resulted in 945,769 reads [NCBI-SRA: SRR1611613] which were used for GenomeZipper calculation. The GenomeZipper pipeline reconstructs gene order based on comparative analysis and synteny conservation with already sequenced grass species. We used *Brachypodium* [28], rice [29] and sorghum [30] genome sequence in this study. The GenomeZipper of 3DS comprises 578 non-redundant reads, which correspond to 498 unique positions on the chromosome arm (see Additional file 6 for complete GenomeZipper data sheet). 567 stringent non-redundant reads with length 504 ± 53 bp were used in this work. The remaining eleven reads had multiple positions in the linear gene order and hence were excluded from the analysis. The second dataset was derived from genetic map of *Aegilops tauschii*. Sequences with length 477 ± 170 bp underlying 7,136 SNPs [47] which mapped to any of the seven *Ae. tauschii* chromosomes were selected and used in this work (see Additional file 7 for list of sequences used).

### Read alignment

Sequence reads of individual BAC pools were aligned using Mosaik 1.1.0021 [48] to both sequence datasets. First, the sequence datasets and paired-end reads from individual BAC pools were converted to binary data format using MosaikBuild. Then, paired-end reads of each BAC pool were mapped to sequence data set using MosaikAligner. “Hash size” (k-mer length for alignment) and “alignment candidate threshold” (minimal alignment length to map the read) parameters were pre-optimized prior to the analysis with respect to alignment sensitivity and time needed for the analysis. To calibrate the parameters, we mapped one row pool (rP) against the GenomeZipper sequence dataset with hash size ranging from 10 to 30, and alignment candidate threshold ranging from 20 to 80. Final alignment was done with the following settings: hash size 20; alignment candidate threshold 40; number of mismatches 3 with the GenomeZipper dataset, and 5 with the *Ae. tauschii* sequence dataset. Number of mismatches should be selected carefully. It strongly depends on divergence of genotypes (or even species) used to produce reads and reference sequence and also on quality of sequence reads and reference used for alignment. For our data, we optimized number of mismatches for GenomeZipper with row pool rP (see Additional file 8). To estimate number of mismatches for *Ae. tauschii* sequence data set we aligned sequences available for bread wheat D genome and *Ae. tauschii* using blastn (data not shown). Non-uniquely mapped reads were resolved and filtered using MosaikSort. Finally, coverage statistics for each sequence in both datasets was calculated using MosaikCoverage. Text files produced by MosaikCoverage were used in further analysis to anchor sequences to BAC clones.

### BAC address deconvolution

A home-made perl script (https://github.com/cvikova/seq_anchoring) was developed for deconvolution of read-mapping information to positive BAC clone(s). For each sequence in both datasets the length of sequence region covered by reads from a particular pool was parsed from MosaikCoverage text files (we call this parameter here after *covered region*). The BAC pool was considered positive for the sequence if its covered region was at least 80% of the sequence length. BAC clone addresses were then calculated from positive pools in three steps. 1) For sequences with only one positive result in each dimension (plate, row and column: 1-1-1) BAC address could be immediately assigned (anchor_type_1). 2) For sequences with more than one positive result in at least one dimension, all candidate BAC addresses were calculated. After that, the file containing the FPC results of the physical map was parsed for the position of BAC clones in contigs. If two (or more) clones, overlapped in the same contig, the particular sequence was assigned to both (all) clones (anchor_type_2). 3) In the third round of analysis, end clones of physical-map contigs were compared in FPC to each other at the cutoff 1e-25 (the physical map was built at 1e-45). If two clones among candidate clones had significant hit, particular sequence was assigned to both of them (anchor_type_3) and corresponding contigs could be merged in the physical map. With respect to the low sequence depth of some DNA pools and the outcome of BAC address deconvolution and PCR validation, we performed the same analysis for decreasing successive covered regions down to 30% of sequence length of each particular sequence.

### PCR validation

PCR reaction contained 1 × PCR buffer, 0.01% Cresol Red, 1.5% sucrose, 0.2 mM each of dNTPs, 0.5 U Taq polymerase, 1 μM primers (for primer list see Additional file 4), 10 ng DNA of particular pool or 0.5 μl of BAC clone DNA (10 – 50 ng/μl). PCR was performed using a C1000 Touch Thermal Cycler (Bio-Rad, Hercules, California, USA) as follows: initial denaturation at 95°C for 5 min; 35 cycles of denaturation at 95°C for 30 sec, annealing (at Ta) for 30 sec and extension at 72°C for 30 sec; final extension at 72°C for 5 min. PCR product were separated on 1.5% agarose gel, stained by ethidium bromide and visualised on IN Genius Syngene Bio Imaging system (Syngene, Cambridge, UK).

## Additional files

Additional file 1:
**BAC pool sequencing and alignment statistics.** Number of reads, estimated sequencing coverage and number of sequences covered by pool reads for GenomeZipper and *Aegilopstauschii* datasets are provided. Additional file 2:
**Final analysis results at successive covered region values from 80 to 30 for both datasets.** Complete list of positive pools and positive BAC clones is provided for each value of covered region and all sequences used in the study. Additional file 3:
**Comparison of anchoring results at different values of covered region for both datasets.** Number of anchored sequences (anchor_type_1, anchor_type_2 and anchor_type_3) and not anchored sequences was compared among individual covered region values representing different stringency of analysis. Highest number of sequences was anchored at less stringent conditions for both datasets. Additional file 4:
**Primers used for anchoring evaluation and resolving questionable clones.**
Additional file 5:
**Complete list of 758 anchored sequences including names of anchored contigs and positive clones.**
Additional file 6:
**GenomeZipper of wheat chromosome arm 3DS.** Virtual gene order was reconstructed based on comparative analysis and synteny conservation with *Brachypodium*, rice and sorghum genomes. “All non red. reads stringent” were used for anchoring. Additional file 7:
**Sequences underlying SNPs genetically mapped in** ***Aegilops tauschii*** **genome.** Chromosome of *Ae.tauschii*, SNP name, genetic position and name of contigs in physical map is provided for each of 7,136 sequences. Additional file 8:
**Test of number of mismatches for read alignment.**
